# Change for the Better: Phosphoregulation of Proteins Drives Evolution

**DOI:** 10.1371/journal.pbio.1000127

**Published:** 2009-06-23

**Authors:** William Mair

**Affiliations:** Freelance Science Writer, La Jolla, California, United States of America

One hundred and fifty years after Charles Darwin proposed that “endless forms” of life, past and present, evolved by natural selection, researchers are still working out what processes generate the variation that natural selection operates on. A primary source of genetic variation arises from spontaneous mutations within the genome, but how these changes give rise to new traits that increase fitness—an organism's ability to pass on its genes to the next generation—remains a central question. “Natural selection works solely by and for the good of each being,” Darwin wrote in *On the Origin of Species*, yet, in the majority of cases, mutations are likely to be detrimental rather than advantageous. Armed with high-throughput genome sequencing tools, researchers today can ask how mutations generate positive outcomes in different species and which types of genes are most likely to change for the better.[Fig pbio-1000127-g001]

**Figure pbio-1000127-g001:**
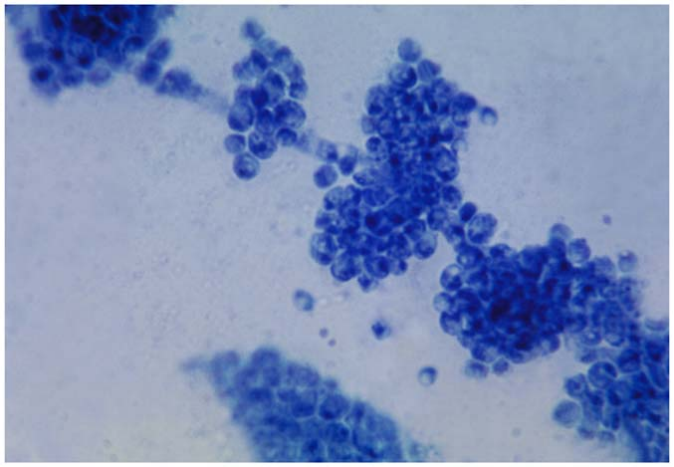
What evolutionary modifications contribute the most to phenotypic diversity? This study by Beltrao et al. takes a look at the evolution of regulation by phosphorylation in three yeast species, *S. cerevisiae*, *C. albicans* (above), and *Sc. pombe*, to show that protein kinases are an important source of variability. (Image credit: CDC/Dr. Godon Roberstad)

One way to tackle these questions is by focusing on how proteins change over time, both at the level of the gene that encodes them and at the level of the protein itself, and how these changes affect basic cell functions. In this issue of *PLoS Biology*, Pedro Beltrao and colleagues describe the use of high-throughput mass spectrometry analysis to compare the phosphorylation status of proteins in three species of yeast. Their results demonstrate how changes to this regulation of protein activity provide a crucial source of variation that might drive the evolution of new phenotypes.

To date, evolutionary biologists largely have focused on transcription factors when looking for instances where genetic mutations might be adaptive rather than disruptive. Transcription factors regulate gene activity by specifically binding to short sequences of DNA. If mutations in these sequences altered the expression of a transcription factor's target genes in a way that increased the fitness of the individual, natural selection would chose the mutant form over the norm.

Whether genes are on or off, however, is far from the whole story when it comes to how organisms function; many proteins must be modified by processes like phosphorylation before they can carry out their function in the cell. Phosphorylation involves enzymes called kinases adding a phosphate group to a specific amino acid within another protein, which creates a negative charge on that amino acid and may, for example, alter its 3-D structure. Phosphorylation, and any subsequent conformational change, is reversible and can either increase or decrease the activity of a protein, for instance, by changing its ability to interact with its substrate.

Just as transcription factors regulate genes via recognizing specific DNA sequences, kinases only phosphorylate proteins that contain particular amino acid motifs. Beltrao et al. therefore decided to study the evolution of phosphoregulation in three related but evolutionarily divergent species of fungi to investigate to what extent kinases might be a source of variation for natural selection to act upon.

To do this, the authors used a high-throughput “shotgun” proteomics approach to identify peptides containing phosphate groups in three species of yeast: *Saccharomyces cerevisiae*, *Candida Albicans*, and *Schizosaccharomyces pombe*. First, they broke down proteins collected from the yeast into fragments using an enzyme that digests them into smaller peptide chunks before separating out only phosphorylated fragments. These fragments were then identified using tandem mass spectrometry, which determines their amino acid sequence based upon their mass.

Since scientists already have an estimate of when the different yeast species diverged, the authors could estimate the speed at which changes in phosphorylation evolved by comparing the differences between the phosphorylated proteins in the three species. Interestingly, the interactions among kinases and their substrate proteins evolved at rates at most two orders of magnitude slower than those of the transcription factors and their target DNA sequences. Beltrao et al. then used previously published as well as novel experimental data to identify proteins that interact with each other genetically and assessed to what extent the different yeast species shared the same interactions. The authors found that genetic interactions involving kinases and transcription factors were less conserved than those involving other proteins, supporting the idea that kinases likely contribute substantially to the evolution of phenotypic diversity.

To expand upon their experimental data, the authors then designed software to predict both the likelihood that a protein will be phosphorylated based upon its amino acid sequence and the kinase that may be responsible. They tested the accuracy of their predictions using proteins that their mass spectrometry had revealed to be differentially phosphorylated among the yeast species. Not only was there a striking overlap between the computational predictions and the experimental data for phosphorylation, but the predicted kinase–substrate interactions generated by the software also matched known interactions. They used this software to predict the phosphorylation status of these proteins in eight additional yeast species, demonstrating just how powerful this tool might be at predicting phospho-regulation computationally.

This new study by Beltrao et al. represents one of the first experimental attempts to quantify proteome-wide evolutionary changes in phosphorylation in different species and suggests that changes to this type of regulation provide a significant source of variation. Natural selection therefore not only acts upon differences at the genetic level but also at the level of protein modification and protein–protein interaction. Rather than proving catastrophic, mutations to kinases and their substrates during evolution may have provided variation that was adaptive, yielding change for the better and perhaps even new species “most wonderful.”

**Beltrao P, Trinidad JC, Fiedler D, Roguev A, Lim WA, et al. (2009) Evolution of Phosphoregulation: Comparison of Phosphorylation Patterns across Yeast Species. doi:10.1371/journal.pbio.1000134**

